# A generalized equation for predicting peak oxygen consumption during treadmill exercise testing: mitigating the bias from total body mass scaling

**DOI:** 10.3389/fcvm.2024.1393363

**Published:** 2024-12-10

**Authors:** Everton J. Santana, Nicholas Cauwenberghs, Bettia E. Celestin, Tatiana Kuznetsova, Christopher Gardner, Ross Arena, Leonard A. Kaminsky, Matthew P. Harber, Euan Ashley, Jeffrey W. Christle, Jonathan Myers, Francois Haddad

**Affiliations:** ^1^ Division of Cardiovascular Medicine, Stanford University School of Medicine, Stanford, CA, United States; ^2^ Stanford Cardiovascular Institute, Stanford University, Stanford, CA, United States; ^3^ Research Unit Hypertension and Cardiovascular Epidemiology, Department of Cardiovascular Sciences, University of Leuven, Leuven, Belgium; ^4^ Division of Pathology, Stanford University School of Medicine, Stanford, CA, United States; ^5^ Department of Medicine, Stanford Prevention Research Center, Stanford University, Stanford, CA, United States; ^6^ Department of Physical Therapy, College of Applied Sciences, University of Illinois at Chicago, Chicago, IL, United States; ^7^ Fisher Institute for Health and Well-Being, Ball State University, Muncie, IN, United States; ^8^ Clinical Exercise Physiology, Ball State University, Muncie, IN, United States; ^9^ Cardiology Division, Veterans Affairs Palo Alto Healthcare System, Palo Alto, CA, United States

**Keywords:** exercise physiology, cardiopulmonary exercise testing, scaling, body composition, oxygen uptake, generalized equation, sex differences, bias

## Abstract

**Background:**

Indexing peak oxygen uptake (VO_2_peak) to total body mass can underestimate cardiorespiratory fitness (CRF) in women, older adults, and individuals with obesity. The primary objective of this multicenter study was to derive and validate a body size-independent scaling metric for VO_2_peak. This metric was termed exercise body mass (EBM).

**Method:**

In a cohort of apparently healthy individuals from the Fitness Registry and the Importance of Exercise National Database, we derived EBM using multivariable log-normal regression analysis. Subsequently, we developed a novel workload (WL) equation based on speed (Sp), fractional grade (fGr), and heart rate reserve (HRR). The generalized equation for VO_2_peak can be expressed as VO_2_peak = Cst × EBM × WL, where Cst is a constant representing the VO_2_peak equivalent of one metabolic equivalent of task. This generalized equation was externally validated using the Stanford exercise testing (SET) dataset.

**Results:**

A total of 5,618 apparently healthy individuals with a respiratory exchange ratio >1.0 (57% men, mean age 44 ± 13 years) were included. The EBM was expressed as Mass (kg)^0.63^ × Height (m)^0.53^ × 1.16 (if a man) × exp (−0.39 × 10^−4^ × age^2^), which was also approximated using simple sex-specific additive equations. Unlike total body mass, EBM provided body size-independent scaling across both sexes and WL categories. The generalized VO_2_peak equation was expressed as 11 × EBM × [2 + Sp (in mph) × (1.06 + 5.22 × fGr) + 0.019 × HRR] and had an *R*^2^ of 0.83, *p* < 0.001. This generalized equation mitigated bias in VO_2_peak estimations across age, sex, and body mass index subgroups and was validated in the SET registry, achieving an *R*^2^ of 0.84 (*p* < 0.001).

**Conclusions:**

We derived a generalized equation for measuring VO_2_peak during treadmill exercise testing using a novel body size-independent scaling metric. This approach significantly reduced biases in CRF estimates across age, sex, and body composition.

## 1 Introduction

**Figure 1:**
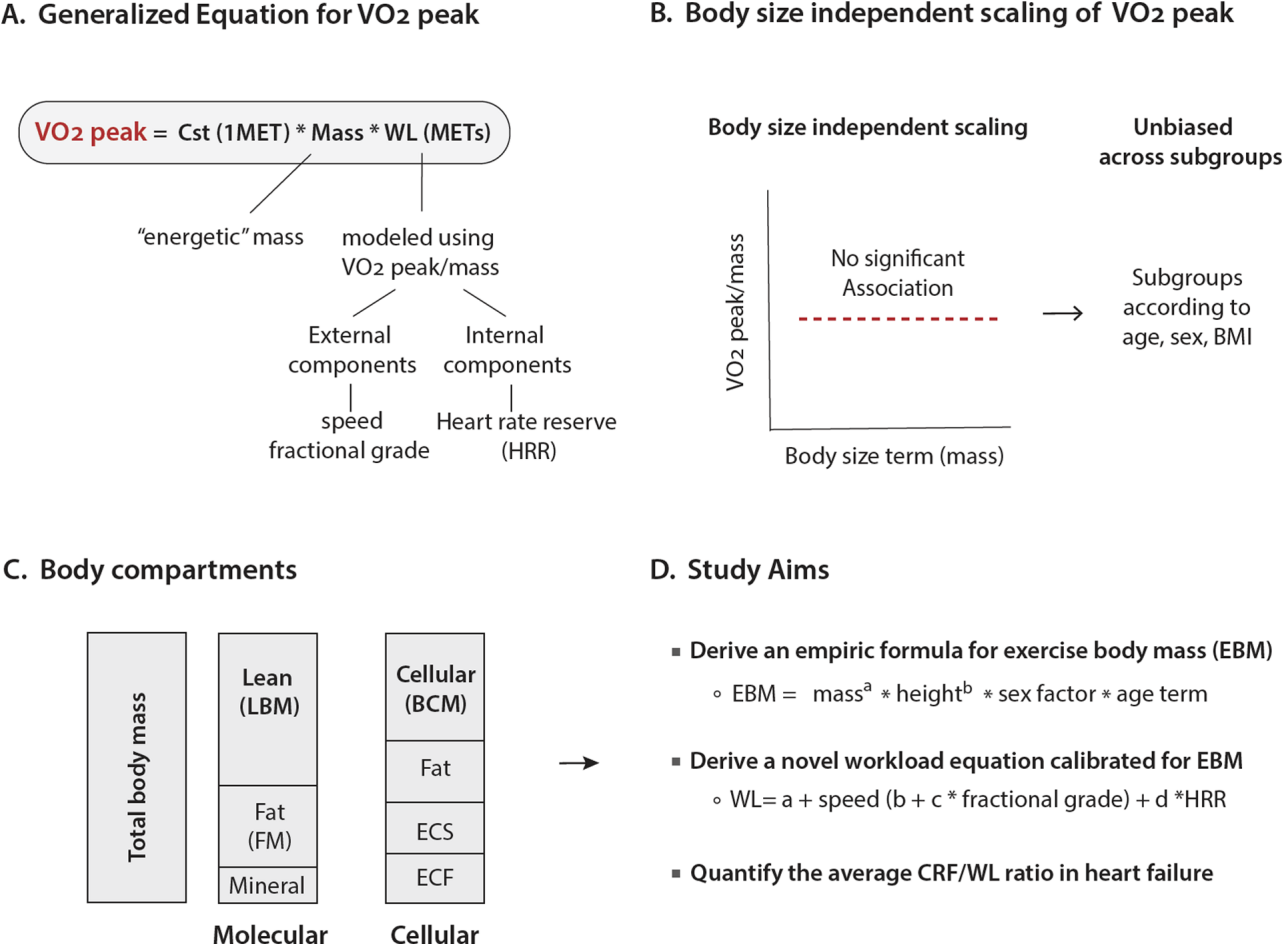
Generalized oxygen consumption equation and body compartments for scaling. **(A)** The generalized equation for peak oxygen consumption with a constant representing the metabolic equivalent at rest; a mass term, which would ideally reflect the energetic mass; and a “workload term,” which reflects absolute exercise intensity. **(B)** Illustrated criteria for body size-independent scaling and calibration among the subgroups. **(C)** Body mass can be assessed using different compartments. LBM measures the specific tissue compartment after the exclusion of the FM and bone mineral content. BCM measures the overall cell mass of the body, usually with K isotope methods. Both LBM and BCM better relate to the metabolically active tissue. **(D)** Summary of the aims of the study.

Cardiorespiratory fitness (CRF) is the capacity of the circulatory and respiratory systems to deliver oxygen to skeletal muscles during physical activity and is widely recognized as one of the strongest predictors of survival in the general population (1–3). In clinical practice, CRF is usually measured by peak oxygen consumption (VO_2_peak), which is typically indexed to total body mass, i.e., VO_2_peak/mass (Figure 1A) (1).

While indexing VO_2_peak to total body mass partially adjusts for body size differences, it does not fully account for variations in metabolically active tissue or differences in body composition. In fact, studies have shown that indexing to total body mass underestimates CRF in women, older adults, and individuals with obesity (4, 5). An ideal metric for scaling VO_2_peak would provide unbiased CRF estimates after accounting for differences in exercise capacity (6). Practically, this is tested by assessing the relationship between indexed VO_2_peak and body size (Figure 1B). To improve VO_2_peak indexing, several approaches have been considered including allometric scaling (body size*^a^*, where *a* is an exponent), the use of different body compartments, or a combination of both approaches. At the molecular level, the body can be divided into lean body mass (LBM), fat mass (FM), and mineral content; at the cellular level, the body is divided into body cell mass (BCM), extracellular solids, extracellular fluids, and fat (Figure 1C) (5, 7). Compared to LBM, BCM better accounts for age-related increases in extracellular solids or fluids, which are generally not metabolically active (5, 7).

Evidence for allometric scaling of total body mass or LBM comes from both animal and clinical studies. In a 1981 study, Taylor et al. found that maximal VO_2_ scales allometrically with body mass, with an exponent of 0.79 (95% CI: 0.75–0.83) in wild animals and 0.77 (95% CI: 0.68–0.85) in domestic animals (8). In a recent meta-analysis of 36 studies involving 6,514 participants, Lolli et al. reported that the pooled allometric exponent for indexed aerobic capacity was 0.70 (95% CI: 0.64–0.76) for total body mass and 0.90 (95% CI: 0.83–0.96) for fat-free mass (9). In the DR's EXTRA study, Krachler et al. also confirmed that VO_2_peak scales more closely to LBM compared to total mass and Kohler et al. showed the potential added benefit of BCM-based scaling (5).

Although the allometric studies showed that scaling to mass^0.70^ provided better body size independence than indexing to total body mass, we hypothesized that including height, sex, and age in the allometric scaling would better account for metabolically active tissue. Therefore, the first objective of this work was to develop and validate a body size-independent scaling metric for VO_2_peak. Here, we introduce a novel metric, referred to as exercise body mass (EBM) that includes mass, height, age, and sex factors (Figure 1D). Building on the EBM concept, our second objective was to develop a well-calibrated workload (WL) equation that not only adjusts for treadmill speed (Sp) and fractional grade (fGr) (10) but also for heart rate reserve (HRR) (11). Integrating the two equations, we derived and validated a generalized VO_2_peak equation expressed as constant × EBM × WL, where the constant represents one metabolic equivalent of task (MET). The calibration of this equation was compared to the standard equation VO_2_peak = 3.5 × mass × WL across the spectrum of age, sex, and body mass index (BMI). Finally, we determined whether the calibration for the generalized equation also holds in patients with heart failure (HF), as these patients often have sarcopenia or frailty (12).

## 2 Materials and methods

### 2.1 Study cohorts

This study included two exercise cohorts, i.e., the Fitness Registry and the Importance of Exercise National Database (FRIEND) registry (13) and the Stanford exercise testing (SET) registry (14). A third cohort, the Diet Intervention Examining the Factors Interacting with Treatment Success (DIETFITS) (15), was used to determine associations between EBM and directly measured LBM.

The FRIEND registry is a multi-institutional initiative established in 2014 with a primary goal of establishing reference CRF values in the United States across the adult life span (13). Cardiopulmonary exercise testing (CPX) laboratories from California, Connecticut, Indiana, Illinois, Louisiana, Maryland, North Carolina, Tennessee, and Texas contributed data to the registry (16). The procedures used for acquiring and managing the FRIEND registry data have been previously reported (17, 18). In brief, all the CPX laboratories contributing data to the FRIEND registry used valid and reliable calibration and testing procedures, and experienced personnel qualified to conduct exercise tests to maximal exertion (17). For the current study, participants who completed a graded exercise treadmill test with a peak respiratory exchange ratio (RER) ≥1.0, age ≥18 and ≤79 years, and BMI between 18.5 and 40 kg · m^−2^ were considered for inclusion. To select an apparently healthy group, participants with cardiovascular disease (CVD), HF, chronic lung disease, chronic kidney disease (CKD), chronic liver disease, endocrine disorders, neurological disorders, or diabetes mellitus were excluded.

The SET registry includes individuals who underwent CPX at Stanford University Medical Center since April 2007. Stanford's CPX personalized ramp is based on an initial assessment of the Veterans Specific Activity Questionnaire (VSAQ) (19). For the validation cohort, we selected two groups of individuals: (1) a lower-risk group without a diagnosis of CVD or HF with an age-predicted CRF >80% according to the Wasserman formula and a minute ventilation/carbon dioxide production (VE/VCO_2_) slope <32 (*n* = 198); and (2) patients with HF or cardiomyopathy (*n* = 1,339).

The DIETFITS was a randomized controlled weight loss trial comprising 609 participants assigned to either healthy low-carbohydrate or healthy low-fat diets (15). Dual-energy x-ray absorptiometry (DXA) scans were performed on 466 participants at the baseline visit using a Hologic QDR-4500 W fan-beam scanner (Bedford, MA, USA) based on the manufacturer's guidelines. A three-compartment model quantifying LBM, fat mass, and bone mineral content was used.

### 2.2 Data collection

For each cohort, data collection included demographics (age, sex, and race), vital signs (blood pressure and heart rate), and anthropomorphic data (height, body mass, and BMI). Exercise variables included Sp, fGr, HRR, and VO_2_peak (measured using open-circuit spirometry). The literature-based WL was calculated using Kokkinos et al.’s formula derived from the FRIEND registry: VO_2_peak = Mass (kg) × [Sp (m/min) × (0.17 + fGr × 0.79) + 3.5] divided by one MET (∼3.5 ml O_2_ · kg^−1^ · min^−1^ (10).

### 2.3 Statistical analyses

The analyses were performed using Python 3.10. Continuous variables were presented as mean and standard deviation (SD) or interquartile range as appropriate and categorical variables as percentages. Upset plots were used to present the prevalence of co-morbidities in the FRIEND registry. For regression analyses, linear multivariable backward-weighted regression was used, in which variables with *p* > 0.05 were removed sequentially. For the log-linear modeling, the natural logarithmic of VO_2_peak or mass and height were used and then transformed to their multiplicative expression.

#### 2.3.1 Deriving EBM to index Vo_2_peak and its body compartment correlates

##### 2.3.1.1 Deriving the EBM terms using a multivariable multiplicative model

The natural logarithm (ln) of VO_2_peak was used as the dependent variable while the independent variables consisted of ln(mass), ln(height), sex, age terms (age, age^2^, and age^3^), Sp, fGr, and HRR. The coefficients obtained in this model were used to express EBM as Mass*^a^* × Height*^b^* × Male factor (e*^c^*) × exp(*d* × Age factor), where *a, b, c,* and *d* are coefficients. Interactions with sex were tested and retained if they significantly differed from a common allometric model. An **approximation** of the allometric EBM equation was also derived using weighted least square regression, where EBM was the dependent variable and mass, height, and age were the independent variables; such expression would be considered valid if it presents an *R*^2^ close to unity and minimal variance.

##### 2.3.1.2 Testing body size independence for EBM scaling of Vo_2_peak

Body size independence for scaling occurs when no significant relationship is observed between an indexed variable and the body size metric. Since VO_2_peak also depends on exercise intensity, we not only tested body size independence in the entire cohort but also in different WL categories and in men and women separately.

##### 2.3.1.3 Biological plausibility of EBM assessment using body compartment correlates

The DIETFITS has an age range of 20–50 years old and allows the testing of mass and height allometric coefficients as well as sex factor in this age range. Using the DIETFITS cohort, we assessed the linear relationship between EBM and LBM^0.9^ with an intercept at the origin. We chose an allometric coefficient of 0.9 for LBM based on the meta-analysis of Lolli et al. (9). To visualize whether EBM could theoretically approximate BCM, we converted EBM to EBM^1.11^ and plotted the resulting values over the age range; EBM was adjusted to a power of 1.11 (the inverse of 0.9) to provide an equivalent non-allometric expression of BCM. In this plot, we superposed the measured BCM values of the study of St-Onge et al. in healthy individuals with a BMI <35 (digitized curves from the original study) (20).

#### 2.3.2 Deriving a novel WL equation based on EBM

##### 2.3.2.1 Deriving a WL equation integrating external and internal factors

Using VO_2_peak/EBM, we derived a WL equation using an additive linear backward regression, entering as independent variables the horizontal (Sp) and vertical (Sp × fGr) components (treadmill external factors) as well as the internal factor of HRR. A constant of 11 was used as the equivalent of 1 MET assuming a height for men of 1.75 m and a BMI of 25 kg · m^−2^ (i.e., 76.6 kg); this constant was derived by assuming 1 MET was equivalent to 3.5 ml · min^−1^ · kg^−1^, dividing by the EBM formula and an age of 20 years old, and rounding to the closest integer (more details in Supplementary Methods).

##### 2.3.2.2 Calibration between CRF and WL among different subgroups according to age, sex, and BMI, and external validation

We tested the calibration of the generalized equation in subgroups according to age, sex, and BMI by evaluating the mean slope between VO_2_peak and the VO_2_peak generalized equation (11 × EBM × WL). Radar plots were used to present the average slope according to age (<40, 40–60, >60 years) and BMI (<25, 25–30, >30 kg · m^−2^) in men and women. A slope of 1 indicates a good calibration; a slope >1 represents a higher VO_2_peak index than the WL estimates of CRF while a slope <1 represents a lower VO_2_peak index than the WL estimates of CRF. The equation was then tested externally in the SET registry in patients without HF and within the CRF range according to the Wasserman formula.

#### 2.3.3 Quantifying the difference in CRF when scaling to total body mass or EBM

We compared the differences between VO_2_peak indexed to total body mass or that indexed to the EBM standard in the men and women (BMI < 25 kg · m^−2^), normal weight and obese (BMI < 25 vs. >30 kg · m^−2^), and younger and older age (<40 vs. >60 years) subgroups. The effect size was measured using relative mean ratios (%) and the Cohen's D effect size. Partition plots were used to visualize VO_2_peak indexed to total body mass or EBM, with values standardized for one MET. For total body mass, the constant used was 3.5 ml · min^−1^ · kg^−1^ and for EBM, the constant was 11.

#### 2.3.4 The CRF/WL ratio in patients with HF

In the patients with HF or cardiomyopathy from the FRIEND registry, the VO_2_peak index may be lower than WL due to the influence of anaerobic metabolism, sarcopenia, or fluid overload. We, therefore, assessed the average slope VO_2_peak/WL relationship in patients with HF. We also validated the average underestimation in the SET registry.

## 3 Results

### 3.1 The FRIEND cohort

**Figure 2:**
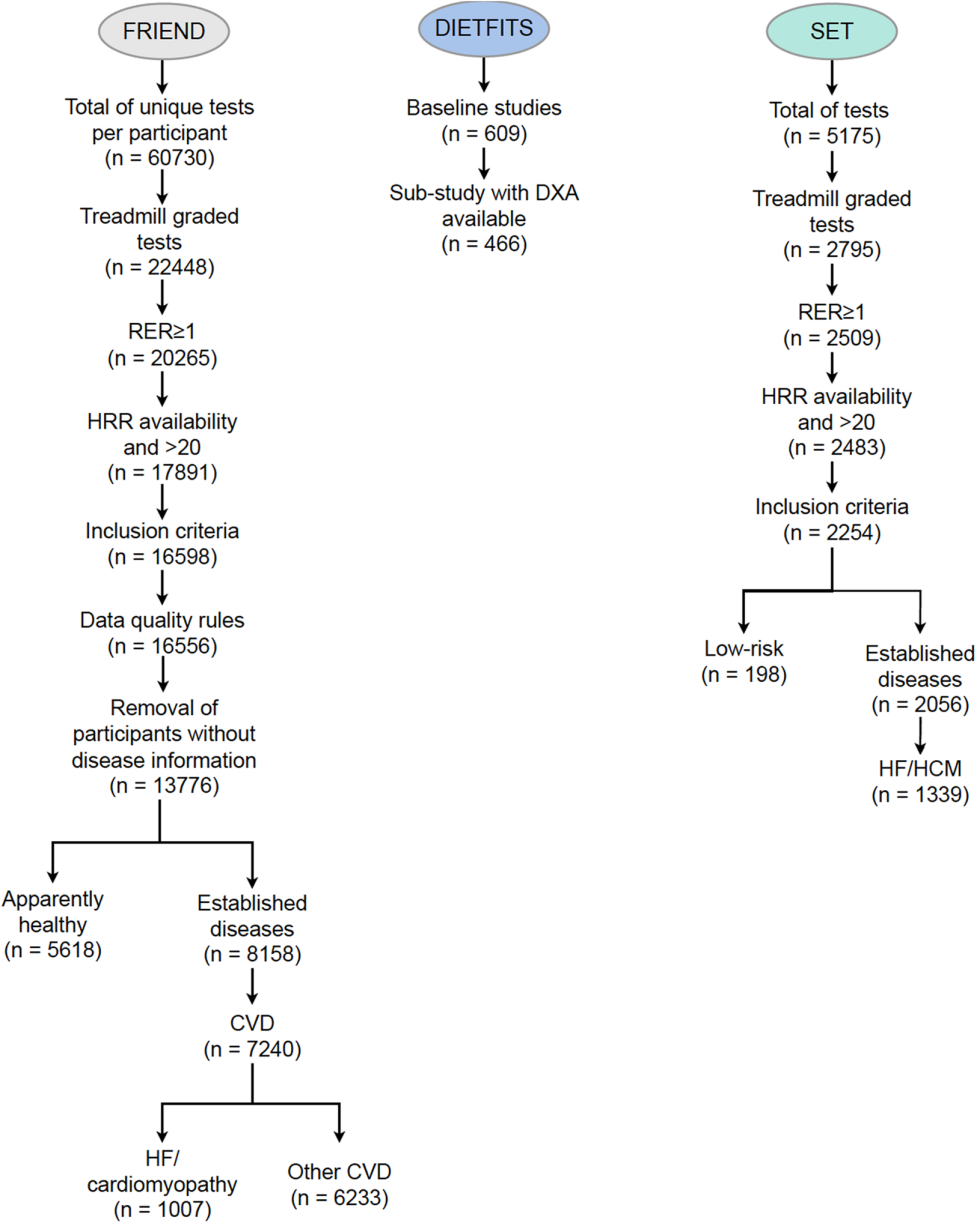
Consort diagram for the FRIEND (derivation cohort), SET (external validation cohort), and DIETFITS (contextualization cohort) datasets. CVD, cardiovascular disease; DIETFITS, Diet Intervention Examining the Factors Interacting with Treatment Success; DXA, dual-energy x-ray absorptiometry; FRIEND, Fitness Registry and the Importance of Exercise National Database; HCM, hypertrophic cardiomyopathy; SET, Stanford exercise testing.

A total of 5,618 individuals met the inclusion criteria for the apparently healthy group (Figure 2) and 7,240 patients with CVD were included, of which 1,007 had a diagnosis of HF. The most common co-morbidities were CVD (52.6%) followed by diabetes mellitus (11.9%) and pulmonary disease (5.2%) (Supplementary Figure S1). The characteristics of the FRIEND cohort are presented in Table 1. In the apparently healthy individuals, the average age was 44 ± 13 years, with 57% men and a mean BMI of 26 ± 4 kg · m^−2^. The mean RER was 1.17 ± 0.10, with a percentage age-predicted heart rate (21) of 100 ± 7% and VO_2_peak of 35.6 ± 10.6 ml · kg^−1^ · min^−1^. When compared to the apparently healthy group, the individuals with CVD were older, had higher BMIs, and had lower VO_2_peak values**.**

**Table 1:** Clinical characteristics of the FRIEND population.

	Apparently healthy			Group with CVD		
Characteristic	Total^a^ (*n* = 5,618)	Men (*n* = 3,214)	Women (*n* = 2,404)	Total^a^ (*n* = 7,240)	Men (*n* = 5,324)	Women (*n* = 1,916)
Demographics and anthropometrics						
Age (years)	44 ± 13	44 ± 12	44 ± 13	57 ± 13	58 ± 13	56 ± 14
Height (m)	1.73 ± 0.10	1.78 ± 0.07	1.65 ± 0.06	1.72 ± 0.09	1.75 ± 0.07	1.63 ± 0.07
Mass (kg)	78 ± 16	84 ± 14	70 ± 14	81 ± 16	84 ± 15	73 ± 15
BMI (kg **·** m^−2^)	26 ± 4	26 ± 4	26 ± 5	28 ± 4	28 ± 4	28 ± 5
Baseline vitals and test measurements						
Max RER	1.17 ± 0.10	1.18 ± 0.10	1.17 ± 0.10	1.15 ± 0.09	1.15 ± 0.09	1.13 ± 0.09
VO_2_peak (ml **·** min^−1^)	2,747 ± 899	3,286 ± 731	2,025 ± 515	2,098 ± 700	2,273 ± 696	1,610 ± 424
VO_2_peak (ml · kg^−1^ **·** min^−1^)	35.6 ± 10.6	40.0 ± 10.0	29.9 ± 8.3	25.9 ± 7.7	27.2 ± 7.9	22.5 ± 6.0
VO_2_peak (METs)	10.2 ± 3.0	11.4 ± 2.9	8.5 ± 2.4	7.4 ± 2.2	7.8 ± 2.3	6.4 ± 1.7
Speed (mph, m · min^−1^)	4.8 ± 1.5 129 ± 41	5.4 ± 1.6 144 ± 42	4.0 ± 1.1 108 ± 30	3.5 ± 1.0 94 ± 27	3.6 ± 1.0 97 ± 27	3.2 ± 0.9 86 ± 24
Fractional grade (%)	0.11 ± 0.05	0.11 ± 0.05	0.12 ± 0.04	0.13 ± 0.03	0.14 ± 0.03	0.12 ± 0.04
Resting SBP (mmHg)	120 ± 14	124 ± 13	115 ± 14	124 ± 17	124 ± 17	125 ± 18
Resting DBP (mmHg)	77 ± 10	80 ± 9	74 ± 10	76 ± 10	76 ± 10	76 ± 10
Max HR (bpm)	176 ± 16	177 ± 15	175 ± 16	142 ± 23	141 ± 23	146 ± 23
HRR (bpm)	108 ± 19	112 ± 17	103 ± 20	71 ± 22	71 ± 22	72 ± 22
% Predicted HR (%)	100.3 ± 7.3	100.9 ± 7.0	99.4 ± 7.6	87.5 ± 12.6	87.0 ± 12.5	89.2 ± 12.6

CVD, cardiovascular disease; DBP, diastolic blood pressure; HR, heart rate; HRR, heart rate reserve; RER, respiratory exchange ratio; SBP, systolic blood pressure.

Where not specified, the value is given as mean ± standard deviation.

^a^ *p* < 0.001 for all characteristics when considering a two-sided *t*-test between total columns for apparently healthy and for CVD.

### 3.2 Deriving EBM for indexing Vo_2_peak and showing its clinical implications

#### 3.2.1 Deriving the EBM equation

**Figure 3:**
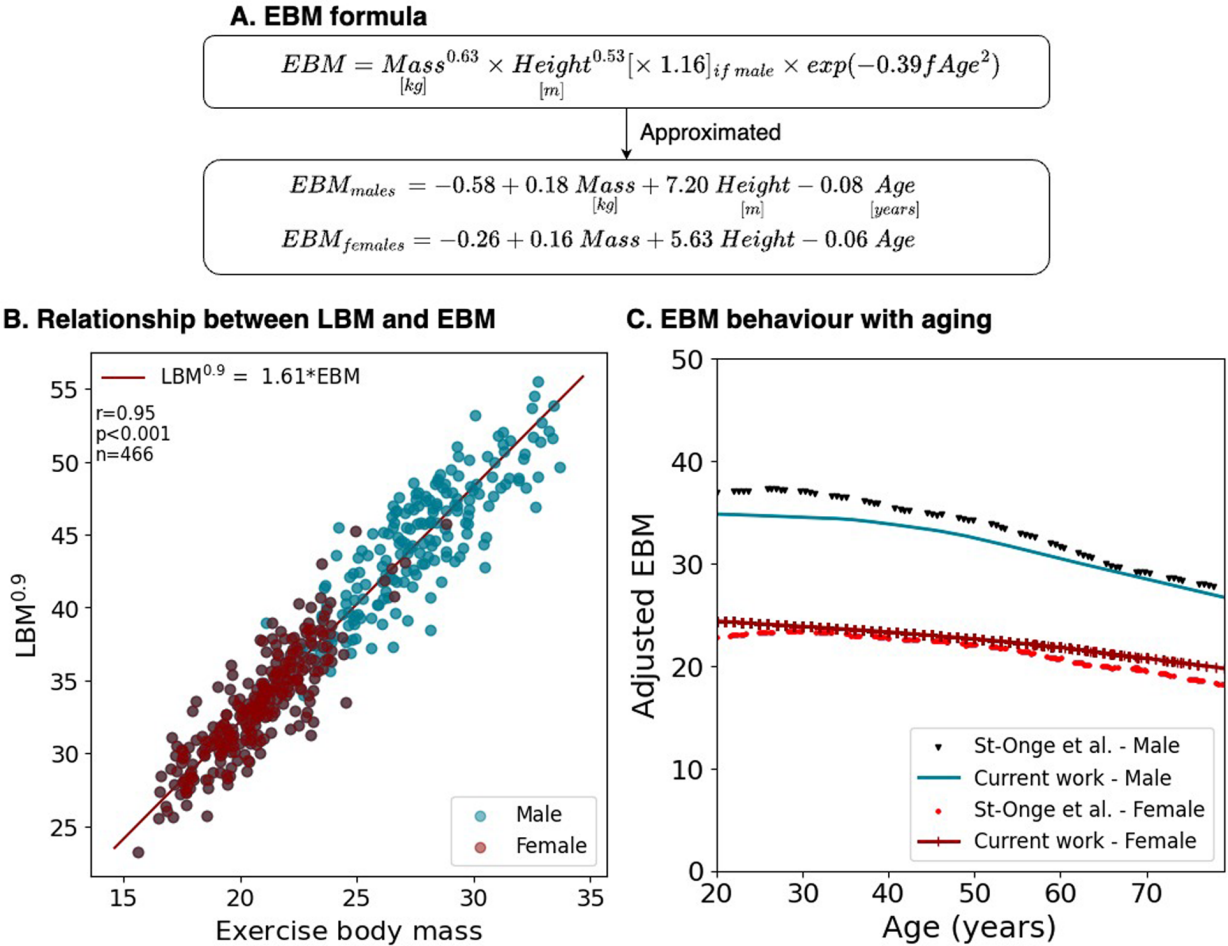
EBM compared to measured values. **(A)** Formulation considering mass and height allometries, sex, and fractional age (fAge = Age/100). **(B)** The relationship between EBM and LBM in DIETFITS. **(C)** Adjusted EBM behavior with aging compared to measured BCM.


The multivariable model allowing the derivation of EBM terms had a coefficient of determination of 0.83, *P* < 0.001, with normally distributed residuals (Supplementary Figure S2). Considering fAge = Age/100, EBM was expressed as (Figure 3):

$$\mathrm{EBM}={\mathrm{Mass}\;(kg^{2})}^{0.63\;}\times {\mathrm{Height}\;(m^{2})}^{0.53}\times 1.16\;(\mathrm{if}\;\mathrm{male})\times \exp\;(-0.39\;\times \;\mathrm{fAg}e^{2})$$

This equation can be approximated to the following additive with a correlation close to 1 and minimal residuals (Supplementary Figure S3):

$$EBM_{males\;\;\;\;\;}=-0.58+0.18\;\mathrm{Mass}\;(\mathrm{kg})+7.20\;\mathrm{Height}\;(m)-0.08\;\mathrm{Age}$$

$$EBM_{females}=-0.26+0.16\;\mathrm{Mass}\;(\mathrm{kg})+5.63\;\mathrm{Height}\;(m)-0.06\;\mathrm{Age}$$

#### 3.2.2 Testing body size independence for EBM indexing of Vo_2_peak

Scaling to EBM provided body size-independent scaling of VO_2_peak with no relationship with EBM (*p* < 0.01) (Table 2). In contrast, scaling to total body mass was significantly associated with the majority of WL intervals in men and women. When compared to scaling to Mass^0.7^, scaling to EBM resulted in lower absolute correlations with several intervals, particularly with 8–8.9 METs for men and with the 6–6.9 and 7–7.9 MET intervals for women.

**Table 2:** Body size independence of VO_2_peak indexed to Mass compared to Mass^0.7^ and EBM in terms of correlation coefficients.

	WL (METs)	*N*	VO_2_peak/Mass	VO_2_peak/Mass^0.7^	VO_2_peak/EBM
Men					
	<7	76	−0.263 (*p* = 0.022)	−0.002 (*p* = 0.988)	0.100 (*p* = 0.389)
	7–7.9	117	**−0.365 (*p*** **<** **0.001)**	−0.128 (*p* = 0.170)	−0.190 (*p* = 0.040)
	8–8.9	296	**−0.168 (*p*** **=** **0.004)**	**0.191 (*p*** **<** **0.001)**	0.150 (*p* = 0.010)
	9–9.9	318	**−0.261 (*p*** **<** **0.001)**	0.065 (*p* = 0.246)	0.049 (*p* = 0.387)
	10–10.9	849	**−0.244 (*p*** **<** **0.001)**	0.069 (*p* = 0.046)	−0.000 (*p* = 1.000)
	11–11.9	418	**−0.285 (*p*** **<** **0.001)**	0.033 (*p* = 0.507)	−0.019 (*p* = 0.701)
	12–12.9	505	**−0.336 (*p*** **<** **0.001)**	−0.061 (*p* = 0.169)	−0.044 (*p* = 0.318)
	13–13.9	256	**−0.306 (*p*** **<** **0.001)**	−0.002 (*p* = 0.974)	−0.076 (*p* = 0.224)
	14–14.9	180	**−0.398 (*p*** **<** **0.001)**	−0.135 (*p* = 0.071)	−0.125 (*p* = 0.096)
	≥ 15	199	**−0.300 (*p*** **<** **0.001)**	−0.013 (*p* = 0.860)	−0.051 (*p* = 0.470)
Women					
	<6	60	−0.292 (*p* = 0.024)	0.031 (*p* = 0.812)	0.097 (*p* = 0.462)
	6–6.9	275	**−0.230 (*p*** **<** **0.001)**	**0.195 (*p*** **=** **0.001)**	0.087 (*p* = 0.148)
	7–7.9	357	**−0.263 (*p*** **<** **0.001)**	**0.154 (*p*** **=** **0.004)**	0.101 (*p* = 0.056)
	8–8.9	601	**−0.327 (*p*** **<** **0.001)**	0.006 (*p* = 0.891)	0.068 (*p* = 0.094)
	9–9.9	310	**−0.403 (*p*** **<** **0.001)**	−0.140 (*p* = 0.014)	−0.012 (*p* = 0.834)
	10–10.9	496	**−0.318 (*p*** **<** **0.001)**	−0.081 (*p* = 0.070)	0.044 (*p* = 0.329)
	11–11.9	139	**−0.284 (*p*** **<** **0.001)**	−0.052 (*p* = 0.546)	0.032 (*p* = 0.706)
	≥ 12	166	**−0.206 (*p*** **=** **0.008)**	0.010 (*p* = 0.903)	0.089 (*p* = 0.254)

*p* < 0.01 are in bold due to multiple comparison.

#### 3.2.3 Biological plausibility of the EBM formula

The characteristics of the DIETFITS cohort are summarized in Supplementary Table S1. The average age was 39 ± 7 years and the BMI ranged from 25 to 40 kg · m^−2^. A strong linear correlation was observed between LBM^0.9^ and EBM with *r* = 0.95, *p* < 0.001, where **LBM^0.9^** = 1.61 · EBM with similar slopes for men and women (Figure 3). To determine whether the age factors in the EBM formula were biologically plausible, we superposed the EBM^1.11^ and BCM values over time from the study of St-Onge et al. (20) using isotope ^40^K (Supplementary Figure S4 for the raw data), as shown in Figure 3. This assumes that BCM would also scale to a power of 0.9 during treadmill exercise.

### 3.3 Deriving a new WL equation based on EBM

#### 3.3.1 Deriving a WL equation integrating external and internal factors

Using multivariable weighted regression, we derived the generalized equation of VO_2_peak expressed as VO_2_peak = 11 × EBM × WL, with the WL term given by

WL(METs)=2+Sp(mph)[1.06+5.22×fGr]+0.019×HRR(bpm).

The overall relationship between the observed and predicted VO_2_peak had an *R*^2^ of 0.85, *p* < 0.001. The WL formula based on EBM yielded, on average, a higher estimated WL than the one derived using Kokkinos et al.’s formula (WL_Kokkinos_ = 0.88·WL_EBM_) with higher values at a lower WL (Supplementary Figure S5).

#### 3.3.2 Calibration between the observed and predicted Vo_2_peak

**Figure 4:**
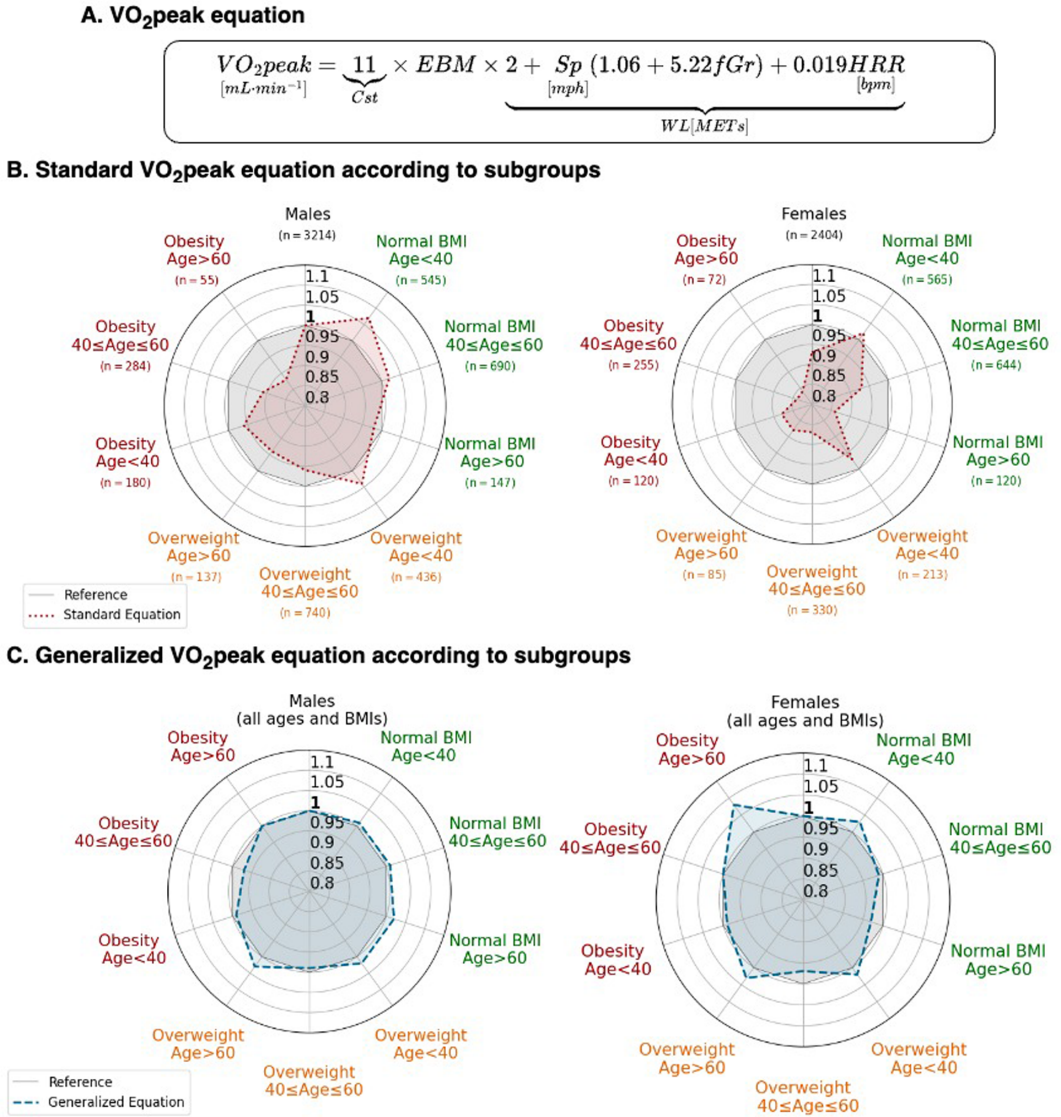
The generalized VO_2_peak equation based upon EBM. **(A)** Formulation considering EBM. **(B)** Predicted VO_2_peak standard equation (red) compared. **(C)** Predicted VO_2_peak allometric equation (blue). For both **(B,C)**, values lower than 1 represent VO_2_peak overestimation by the equation, and greater than 1, an underestimation. The gray reference zone corresponds to a slope of 1. fGr, fractional grade; HRR, heart rate reserve; Sp, speed in mph.

Radar plots show the average calibration slope stratified according to the age, sex, and BMI subgroups for total body mass and EBM standards (Figure 4). These demonstrate better calibration when using the EBM-based generalized equations. Underestimation of CRF using VO_2_peak/total body mass for a given WL was worst (*p* < 0.001) in women, in older individuals, and in individuals with obesity.

#### 3.3.3 Validation in the SET registry

**Figure 5:**
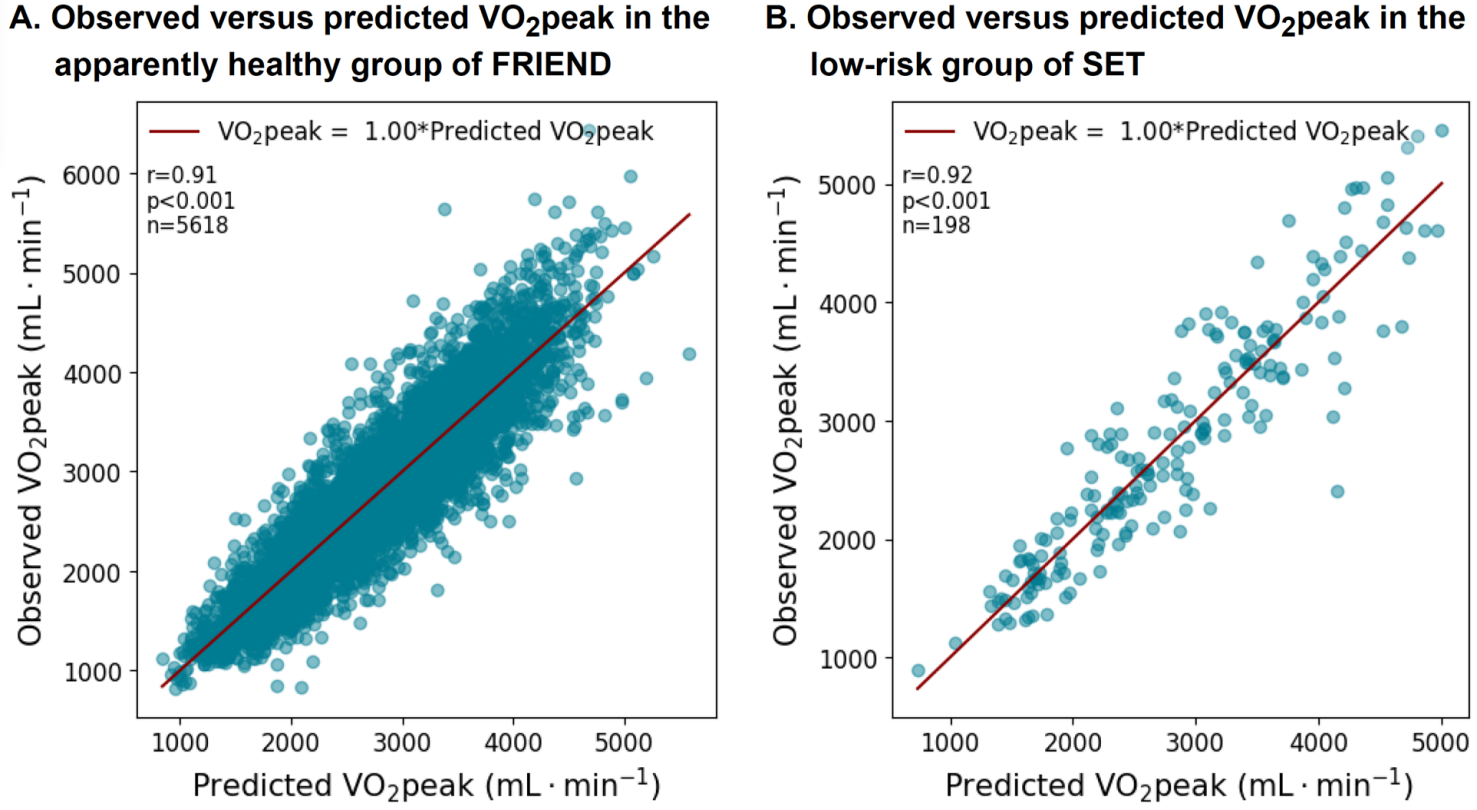
VO_2_peak equation performance in the apparently healthy subgroups from the FRIEND (derivation) and the SET registries (validation).

The characteristics of the patients in the SET registry are presented in Supplementary Table S3. The equation was well-validated in the SET registry with an average slope of 1.0 (*r* = 0.92, *p* < 0.001) (Figures 5A,B). The subgroup analyses by sex, age, and BMI also demonstrate a well-calibrated equation (Supplementary Table S4).

### 3.4 Quantifying the difference in CRF when using the total body mass or EBM standard

**Figure 6:**
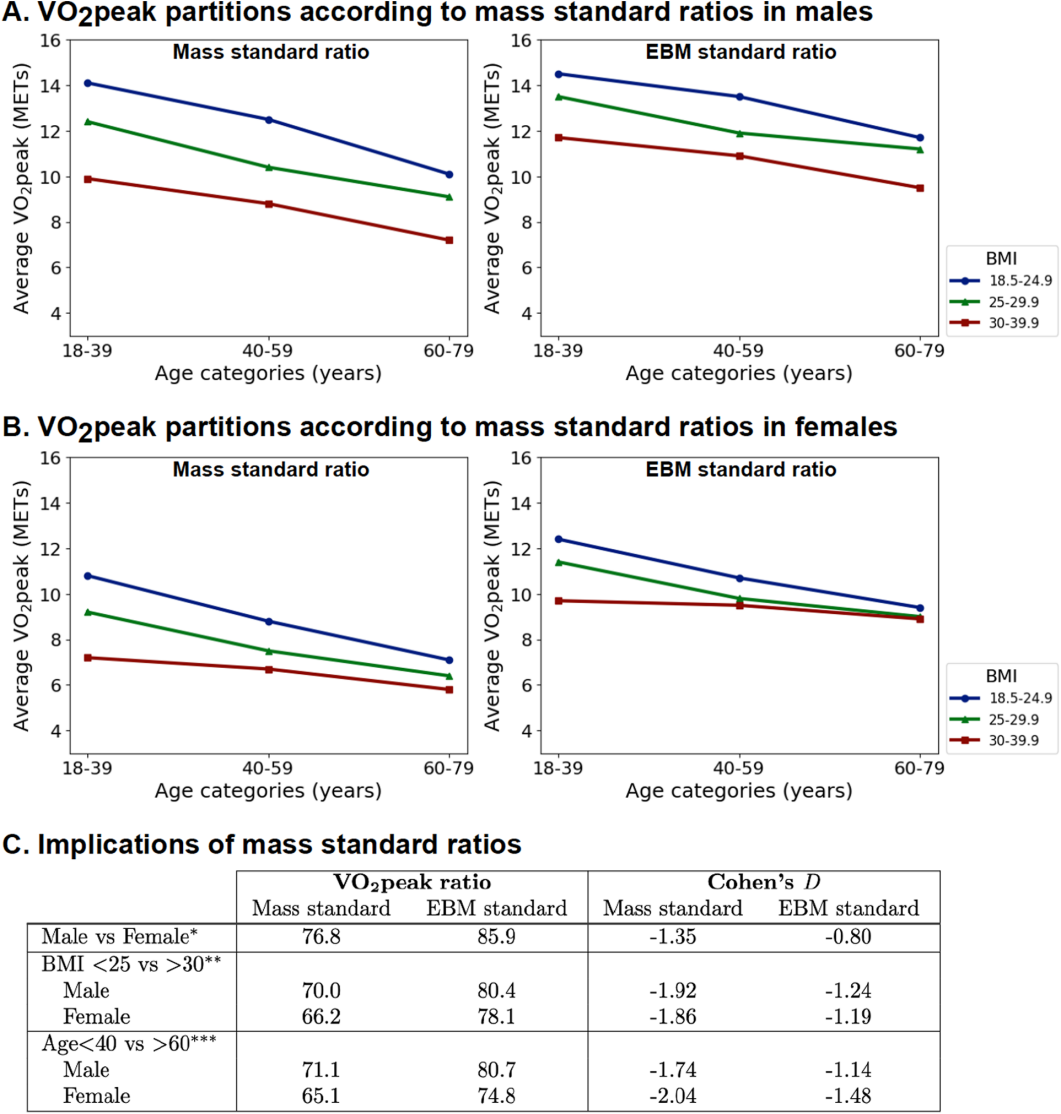
Indexing VO_2_peak to EBM instead of mass reduces age, sex, and BMI differences. **(A)** Partition analysis for men. **(B)** Partition analysis for women. **(C)** Comparison of mean VO_2_peak ratios and Cohen's D effect size (*BMI < 25 and age <40 years; **age <40 years; ***BMI < 25; comparison of all subgroups in Supplementary Figure S5).

The partition plots of the VO_2_peak index for total mass or EBM (presented as METs) are presented in Figure 6. Smaller differences between men and women, older and younger, and obese and normal individuals were observed when using the EBM standard compared to the total mass standard (Figure 6, Supplementary Figure S6).

### 3.5 The CRF/WL relationship in patients with HF

**Figure 7:**
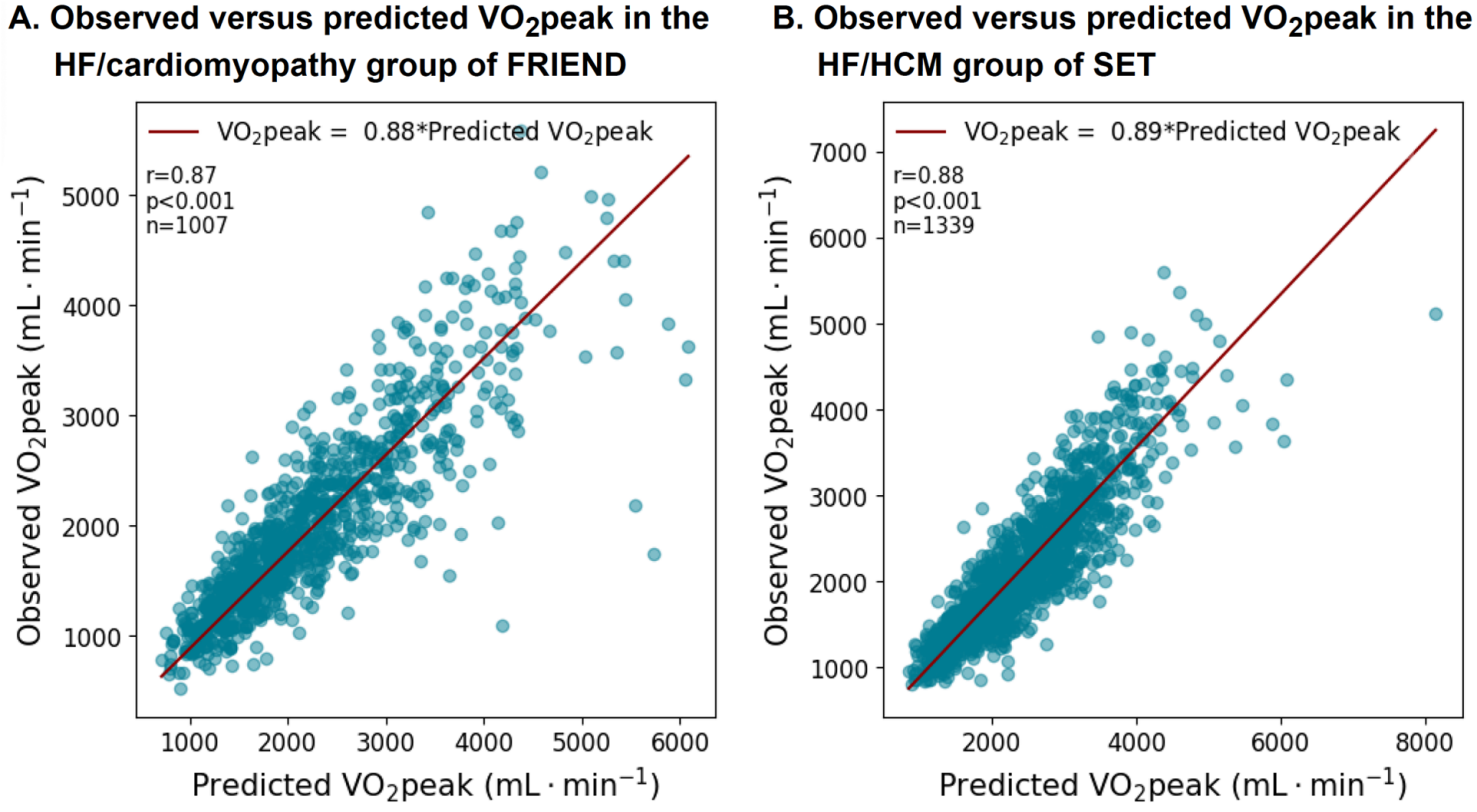
VO_2_peak equation performance in the patients with HF subgroups in the FRIEND (derivation) and SET registries (validation). The VO_2_peak to workload ratio was on average lower in the patients with HF.

In both the FRIEND and SET registries, patients with HF had on average a lower slope compared to apparently healthy individuals as presented in Figure 7 and Supplementary Table S2. Supplementary Table S5 summarizes the main equations related to this work.

## 4 Discussion

In this study, we developed a body size-independent scaling factor for VO_2_peak. EBM provided body size-independent scaling with robust calibration across different ages, sexes, and BMI categories. This has important implications for accurate reporting of CRF in the general population. In addition, our novel WL equation incorporates HRR, which can provide better estimates of energy expenditure during treadmill exercise testing. Finally, we found that in patients with HF, VO_2_peak was often lower than the WL-predicted values, which may be due to an increased reliance on anaerobic metabolism, the presence of sarcopenia, or fluid overload.

Scaling plays a central role in comparative biology as it reduces differences associated with body size and composition (6). This is particularly relevant when comparing men and women, younger and older individuals, or individuals with normal weight and obesity. Several studies have shown that VO_2_peak scales to body size according to a log-linear relationship, providing the basis for allometric scaling (6, 8, 9, 22). In 1981, Taylor et al. conducted a landmark study to determine whether VO_2_max is scaled proportionally to mass (8). Their study included both domestic and wild animals spanning several orders of magnitude of mass (7.2 g–263 kg) and testing for VO_2_max following training sessions in all the animals. They found that VO_2_max scaled to 0.79 (0.75–0.83) in wild animals and to 0.77 (0.68–0.85) in domestic animals. In a recent meta-analysis of 36 studies involving 6,514 participants, Lolli et al. found that the pooled allometric exponent for indexed aerobic capacity was 0.70 (95% Cl of 0.64–0.76) for whole body mass and 0.90 (95% Cl of 0.83–0.96) for fat-free mass (9). A similar mass coefficient of 0.71 was also found in groups of prepubertal, circumpubertal, and adult subjects after accounting for differences in height (23).

Original to our study, we validated, in a large cohort, a body size-independent scaling metric for VO_2_peak. EBM was closely related to LBM and followed the age-associated decrease in BCM described by in previous works (20, 24). Scaling to EBM attenuated differences in VO_2_peak associated with sex, age, or obesity. Scaling to Mass^0.7^ also yielded overall good body size-independent scaling but had the disadvantage of not considering stature or integrating sex differences in body composition. In addition, when scaling to EBM, the sex gap in younger individuals of approximately 10%–15% was consistent with performance difference reported in athletes (25). Using the simplified additive equation for EBM will likely facilitate clinical adoption and have a similar expression as the national health and nutrition examination survey (NHANES) equations for LBM (26). The second original contribution of our study was the development of a WL equation that incorporates HRR; this will provide more personalized estimates of aerobic capacity (11). Compared to the Kokkinos equation (10), the new WL equation provides higher estimates at lower Sp and fGr, reflecting the reduced efficiency of slower locomotion (27–29). This explains why the intercept of the equation was likely higher than unity with a higher early increment.

The goal underpinning the generalized equation is to improve the consistency of CRF reporting across the general population. For instance, at the same Sp, fGr, or HRR, apparently healthy individuals will, on average, have similar aerobic capacity regardless of age, sex, or BMI. The EBM equation is an average scaling tool and not a substitute for direct measurements of LBM or BCM. Specific considerations are needed for trained athletes and frail individuals with sarcopenia: athletes generally have a higher proportion of BCM, while frail individuals with sarcopenia have a lower proportion (30, 31). Without these adjustments, CRF may be overestimated in athletes and underestimated in individuals with sarcopenia. The lower calibration slope observed in patients with HF from both the FRIEND and SET registries could be due to variations in body composition (such as sarcopenia or fluid overload) or increased reliance on anaerobic metabolism. Tanabe et al. (32) previously reported a discrepancy in measured and estimated VO_2_peak (or WL) in patients with HF using an ergocycle (32). Although we were able to confirm the 20% higher estimates, these were reduced to 10% differences after adjusting for HRR.

While our study is the largest to empirically validate a scaling metric for VO_2_peak, several limitations need to be highlighted. First, the EBM formula is based on a United States population of mainly white participants and further adjustment for different races and ethnicity will need to be studied. Second, we did not account for duration of exercise or holding the handrails of the treadmill in our WL estimates. Third, other factors such as peak blood pressure, environment, and diurnal rhythm can also influence VO_2_peak and will require further study. We were also not able to compare EBM to measure LBM in the FRIEND cohort itself due to a lack of DXA, requiring another cohort (DIETFITS) for this task. In addition, the developed equations are specific for treadmill CPX. Finally, future refinements in an EBM formula should consider correction factors for athletic status and sarcopenia/frailty.

In conclusion, the EBM formula enables body size-independent scaling of VO_2_peak and more consistent and equitable reporting of CRF. Incorporating HRR in the WL equations also has the added advantage of a more personalized estimate of aerobic capacity during exercise.

## Data Availability

The datasets presented in this article are not readily available because of ethical restrictions. Requests to access the datasets should be directed to Francois Haddad, fhaddad@stanford.edu.
