# Supplementary material for: A generalized equation for predicting peak oxygen consumption during treadmill exercise testing: mitigating the bias from total body mass scaling
Source: Front Cardiovasc Med. 2024 Dec 10;11:1393363. doi: 10.3389/fcvm.2024.1393363 (PMC11666446; doi:10.3389/fcvm.2024.1393363)
Supplement: Supplementary file 1 [file Datasheet1.pdf]

Supplementary Material

METHODS

For the generalized equation, a constant of 11 was used as the equivalent of 1 MET assuming:

1) 1 MET equivalent of 3.5 $mL\cdot min^{-1}\cdot kg^{-1}$;
2) Height for male of 1.75 m and a BMI of 25 $kg\cdot m^{-2}$ (i.e., 76.6 kg);
3) Age of 20 years-old (as we want an early adulthood reference), and;
4) Rounded to the closest integer.

Thus, for someone with 76.6kg, the equivalent VO_2peak is 3.5 * 76.6 = 268.1 $mL\cdot min^{-1}$.

Since with the generalized equation VO_2peak = Cst * EBM * WL, for the WL = 1 MET, Cst = 268.1/EBM, then:

C**st** = 268.1/ [$76.6^{0.63}$ * $1.75^{0.53}$ * 1.16 * exp (-0.39 *$(20/100)^2$) = 11.34 ≈ 11

FIGURES

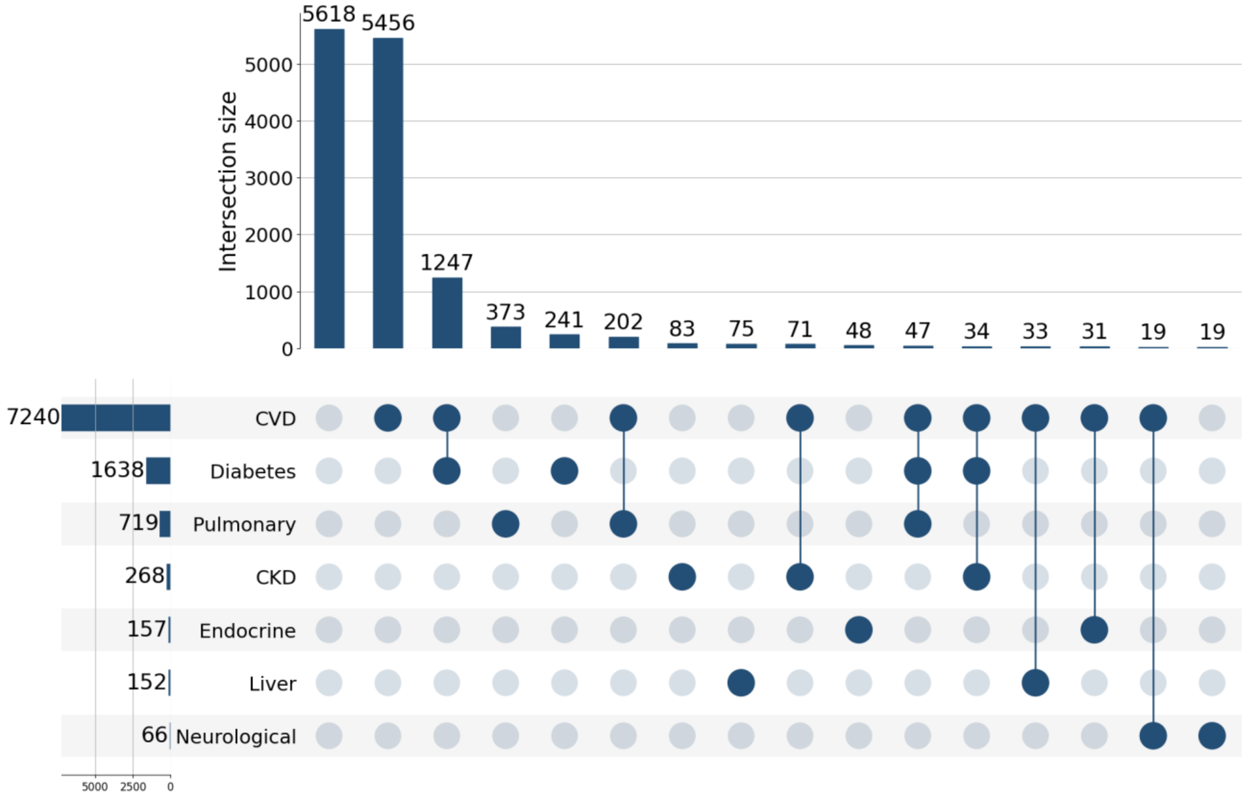

Supplementary Figure 1: *Upset plot showing the distribution of individuals with known diseases in the FRIEND study sample. Only group sizes equal to or greater than 19 are shown. The prevalence of each disease is seen on the left. The first column bar indicates the number of participants who did not have any of the considered diseases (no filled circles), while the other bars indicate unique disease combinations (isolated disease: one filled circle; concomitant disease: two or more filled circles). CVD indicates cardiovascular disease; CKD chronic kidney disease.*

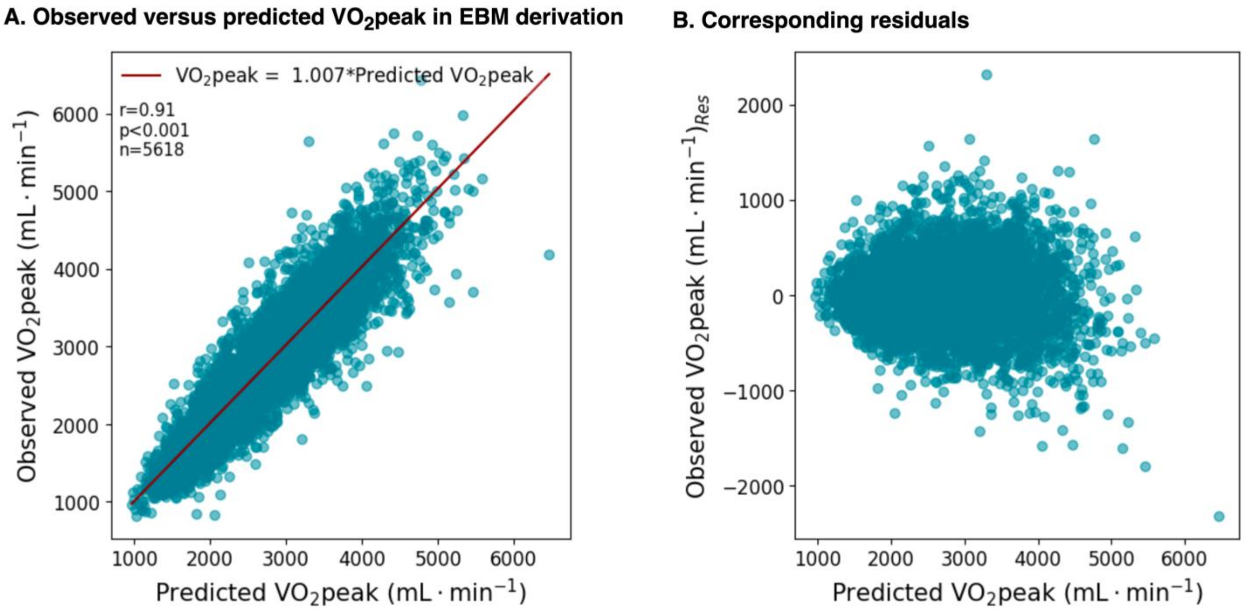

Supplementary Figure 2: *Observed versus predicted VO_2peak during EBM derivation and its residuals.*

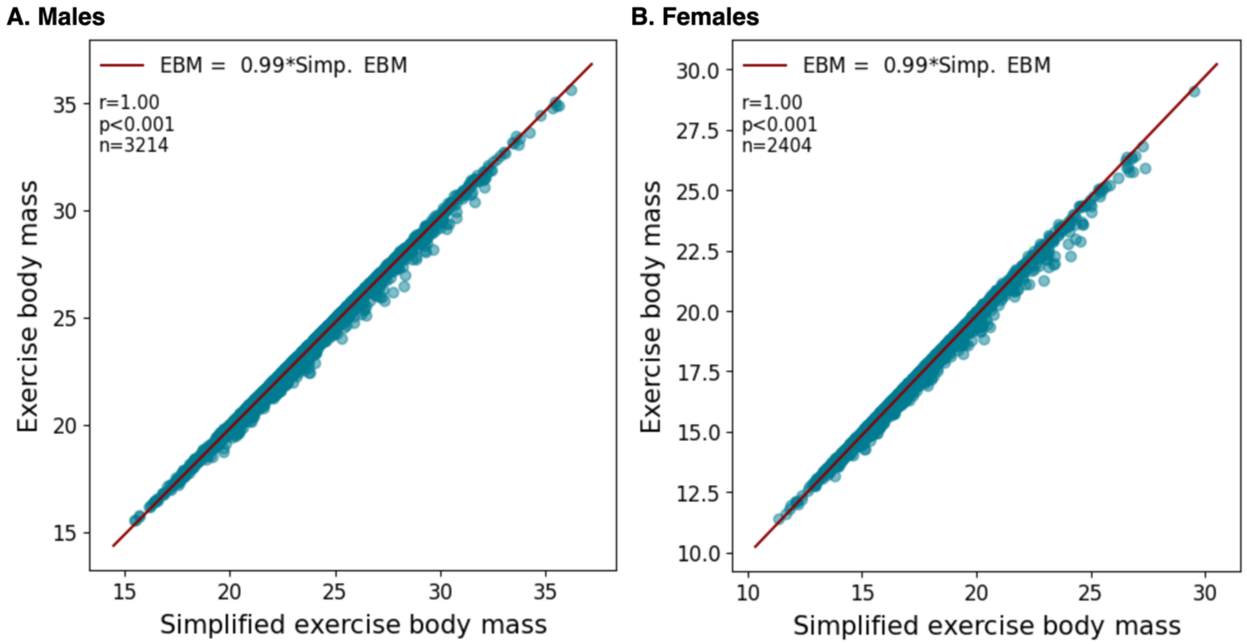

Supplementary Figure 3: *Relationship between exercise body mass equation and its simplified version in the apparently healthy cohort.*

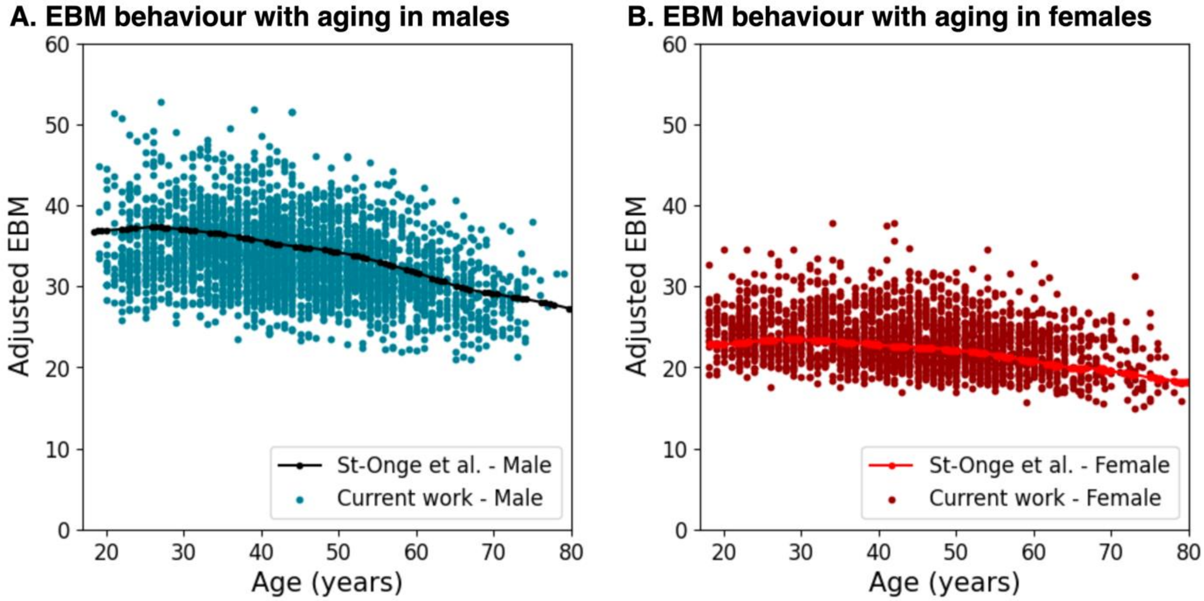

Supplementary Figure 4: *Adjusted EBM behavior with aging compared to measured BCM.*

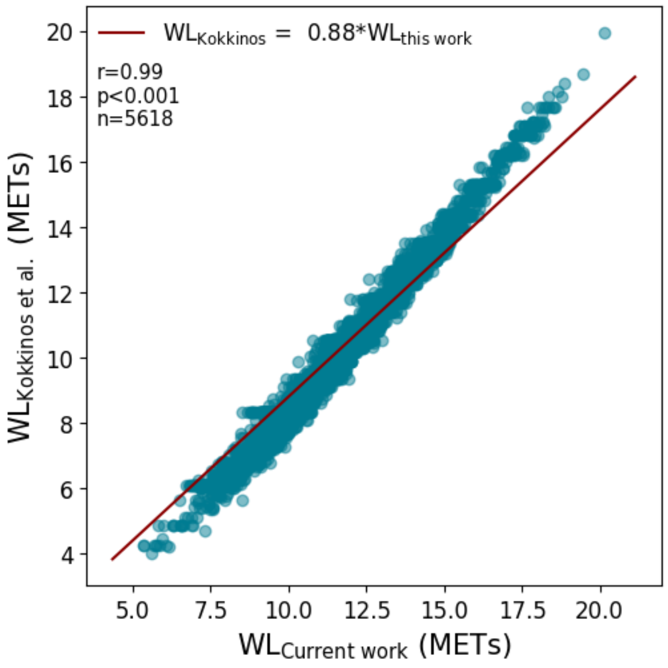

Supplementary Figure 5*: Workload (WL) component of the VO_2peak generalized equation compared with the literature standard equation (Kokkinos et al. equation)*[1].

	**VO_2peak ratio**		**Cohen's D**	
	Mass standard	EBM standard	Mass standard	EBM standard
Sex analysis (male vs female) for BMI<25				
Age<40	76.8	85.9	-1.35	-0.80
40-60	70.4	79.4	-1.61	-1.13
>60	70.3	79.7	-1.72	-1.17
BMI analysis (BMI<25 vs BMI>30) for males				
Age<40	70.0	80.4	-1.92	-1.24
40-60	70.6	80.6	-1.63	-1.10
>60	70.4	80.2	-1.66	-1.12
BMI analysis (BMI<25 vs BMI>30) for females				
Age <40	66.2	78.1	-1.86	-1.19
40-60	75.6	88.3	-1.40	-0.67
>60	82.5	95.8	-1.02	-0.24
Age analysis (Age<40 vs Age>60)				
Male	71.1	80.7	-1.74	-1.14
Female	65.1	74.8	-2.04	-1.48

Supplementary Figure 6: *Comparison of mean VO_2peak ratios and Cohen's D effect size (Figure 4C complement).*

TABLES

***Supplementary Table 1**: Clinical characteristics of the study DIETFITS cohort. Where not specified, the value is given as mean ± standard deviation. DPB: diastolic blood pressure; HDL: high-density lipoprotein; LBM: lean body mass; LDL: low-density lipoprotein; SPB: systolic blood pressure.*

	DIETFITS		
**Characteristic**	**Total (n=466)**	**Male (n=190)**	**Female (n=276)**
**Demographic and anthropometrics**			
Age (years)	39 ± 7	39 ± 7	39 ± 7
Height (m)	1.69 ± 0.09	1.77 ± 0.07	1.64 ± 0.06
Mass (kg)	96 ± 15	105 ± 13	89 ± 12
BMI ($kg \cdot m^{-2}$)	33 ± 3	33 ± 3	33 ± 3
**DXA**			
Fat mass (%)	36.4 ± 6.7	30.1 ± 04.6	40.7 ± 4.0
Fat mass (kg)	33.8 ± 7.4	31.0 ± 7.3	35.7 ± 6.9
LBM (kg)	56.9 ± 11.5	68.1 ± 7.4	49.2 ± 6.1
**Baseline vitals**			
SBP (mmHg)	123.4 ± 12.5	128.1 ± 11.3	120.1 ± 12.2
DBP (mmHg)	81.1 ± 7.5	83.6 ± 7.2	79.4 ± 7.3
Heart rate (bpm)	70.4 ± 10.1	68.3 ± 10.2	71.9 ± 9.9
**Laboratory**			
LDL cholesterol ($mg \cdot dL^{-1}$)	112.4 ± 27.3	115.1 ± 27.9	110.5 ± 26.7
HDL cholesterol ($mg \cdot dL^{-1}$)	49.0 ± 9.8	44.5 ± 7.6	52.1 ± 9.9
Triglyceride ($mg \cdot dL^{-1}$)	128.3 ± 90.7	147.1 ± 73.9	115.4 ± 98.7

Supplementary Table 2*: VO_2peak equation performance in CVD group of FRIEND registry.*

Group	n	r	Slope	Average percentage error ± SD
CVD	7240	0.85	0.94 ± 0.16	0.63 ± 22.17
HF or cardiomyopathy	1007	0.87	0.88 ± 0.18	0.02 ± 22.02
CVD excluding HF and cardiomyopathy	6233	0.85	0.95 ± 0.16	0.73 ± 22.02

Supplementary Table 3*: Clinical characteristics of the study in low-risk and heart failure (HF)/hypertrophic cardiomyopathy (HCM) groups of SET. Where not specified, the value is given as mean ± standard deviation.*

	**Low-risk**			**HF/HCM**		
**Characteristic**	**Total (n=198)**	**Male (n=116)**	**Female (n=82)**	**Total (n=1339)**	**Male (n=445)**	**Female (n=894)**
**Demographic and Anthropometrics**						
Age (years)	46 ± 17	47 ± 17	44 ± 18	50 ± 15	50 ± 15	50 ± 15
Height (m)	1.74 ± 0.11	1.8 ± 0.09	1.66 ± 0.08	1.74 ± 0.1	1.64 ± 0.07	1.78 ± 0.08
Mass (kg)	77 ± 16	83 ± 15	69 ± 13	82 ± 17	72 ± 14	87 ± 15
BMI ($kg \cdot m^{-2}$)	25 ± 4	26 ± 4	25 ± 4	27 ± 4	27 ± 5	27 ± 4
**Baseline vitals and test measurements**						
Max RER	1.15 ± 0.08	1.17 ± 0.08	1.12 ± 0.07	1.13 ± 0.08	1.11 ± 0.07	1.14 ± 0.08
VO_2peak ($mL \cdot min^{-1}$)	2829 ± 1015	3265 ± 938	2213 ± 775	2157 ± 800	1629 ± 459	2420 ± 804
VO_2peak ($mL \cdot kg^{-1} \cdot min^{-1}$)	37.2 ± 12.5	40.1 ± 12.2	33.0 ± 11.9	26.7 ± 9.5	23.1 ± 6.8	28.4 ± 10.1
VO_2peak (METs)	10.6 ± 3.6	11.4 ± 3.5	9.4 ± 3.4	7.6 ± 2.7	6.6 ± 1.9	8.1 ± 2.9
Speed (mph, $m \cdot min^{-1}$)	4.6 ± 1.3	4.8 ± 1.2	4.3 ± 1.3	3.8 ± 1.0	3.5 ± 0.7	4.0 ± 1.1
Fractional grade	0.15 ± 0.04	0.15 ± 0.04	0.14 ± 0.04	0.13 ± 0.05	0.12 ± 0.05	0.13 ± 0.05
Max HR (bpm)	164 ± 18	164 ± 18	164 ± 18	145 ± 26	144 ± 24	146 ± 27
% Predicted HR (%)	93.0 ± 7.3	93.6 ± 7.6	92.1 ± 6.7	83.6 ± 12.4	83.0 ± 11.8	83.9 ± 12.7

Supplementary Table 4: *VO_2peak equation with mass allometry and adjusted for HRR and age in the low-risk group of SET.*

	**n**	**Slope ± SD**	**Average residuals ± SD**	**Average percentage error ± SD**
General	198	1.00 ± 0.13	4.96 ± 389.16	1.37 ± 14.19
Sex Subgroups				
Male	116	1.00 ± 0.11	-3.33 ± 373.60	1.46 ± 11.65
Female	82	1.01 ± 0.18	15.27 ± 412.24	1.36 ± 17.27
Age Subgroups				
Age<40	76	1.01 ± 0.13	9.87 ± 470.53	1.35 ± 15.20
40≤Age≤60	74	1.00 ± 0.13	-0.43 ± 348.25	1.68 ± 12.83
Age>60	48	0.99 ± 0.14	24.91 ± 298.60	-0.60 ± 14.56
BMI Subgroups				
Normal BMI	107	1.03 ± 0.15	6.20 ± 442.28	1.69 ± 16.56
Overweight	69	0.98 ± 0.10	-0.08 ± 308.12	1.05 ± 10.38
Obesity	22	0.96 ± 0.10	9.28 ± 273.56	0.63 ± 12.03

Supplementary Table 5: Summary of the main equations related to this work.

Standard equation
**VO_2peak (mL $O_2 \cdot kg^{-1} \cdot min^{-1}$) = 3.5 * Mass * WL**<br>1 MET = 3.5 mL $O_2 \cdot kg^{-1} \cdot min^{-1}$
**WL (Kokkinos et al.)** = estimated VO_2peak<br>WL (mL $O_2 \cdot kg^{-1} \cdot min^{-1}$) = 3.5 + Sp [$m \cdot min^{-1}$] * (0.17 + 0.79 * fGr)<br>WL (METs) = 1+ Sp [$m \cdot min^{-1}$] * (0.048 + 0.226 * fGr)<br>= 1+ Sp [mph] * (1.29 + 6.06 * fGr)<br>= 1+ Sp [kph] * (0.8 + 3.77 * fGr)
Novel Generalized VO_2peak equation
**VO_2peak (mL $O_2 \cdot min^{-1}$) = 11 * EBM * WL**
**EMB** = Mass(kg)$^{0.63}$ * Height(m)$^{0.53}$ * 1.16 (if male) * exp (-0.39 * fAge^2)<br>Simplified additive equations:<br>$\mathrm{EBM}_{\mathrm{male}}$ = -0.58 + 0.18 Mass(kg) + 7.20 Height(m) - 0.08 Age<br>$\mathrm{EBM}_{\mathrm{females}}$ = -0.26 + 0.16 Mass(kg) + 5.63 Height(m) - 0.06 Ag
WL (current work)<br>**WL (METs)** = 2 + Sp [mph] (1.06 + 5.22*fGr) + 0.019*HRR<br>= 2 + Sp [$m \cdot min^{-1}$] (0.0395+ 0.1946 *fGr) + 0.019*HRR<br>= 2 + Sp [kph](0.66+ 3.24*fGr) + 0.019*HRR (average HRR in FRIEND: 109 bpm)
